# Supplementary material for: Study on the Effect of Asphalt Static Conditions on the Tensile Properties of Acidic Aggregate Hydraulic Asphalt Concrete
Source: Materials (Basel). 2024 May 29;17(11):2627. doi: 10.3390/ma17112627 (PMC11173427; doi:10.3390/ma17112627)
Supplement: Supplementary file 1 [file materials-17-02627-s001.zip › materials-2937682-supplementary.pdf]

-Supplementary Information-

Date: December 25, 2023

Submitted to: *Case Studies in Construction Materials*

Study on the Effect of Asphalt Static Conditions on the Tensile Properties of Acidic Aggregate Hydraulic Asphalt Concrete

Lei Bao [1,2], Min He [1,3,*], Shu Wang [2] and Xinshuang Wu [2]

1 School of Civil Engineering and Architecture, Xi'an University of Technology, Xi'an 710048, China; lbao1013@163.com
2 Power China Northwest Engineering Corporation Limited, Xi'an 710065, China; 13102213129@163.com (S.W.); wuxins@nwh.cn (X.W.)
3 State Key Laboratory of Eco-Hydraulics in Northwest Arid Region of China, Xi'an University of Technology, Xi'an 710048, China
* Correspondence: hem@xaut.edu.cn; Tel.: +86-13309201657

7 Pages with 5 Figures and 5 Tables

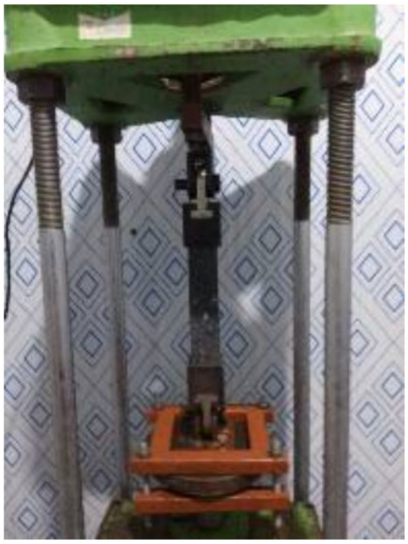

Fig.S1. Tensile Test Apparatus.

(a)

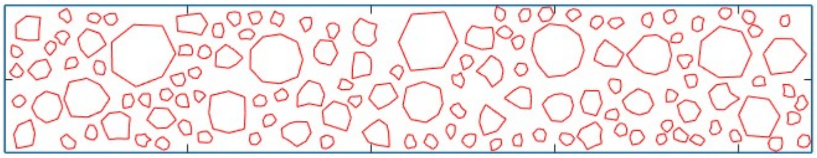

(b)

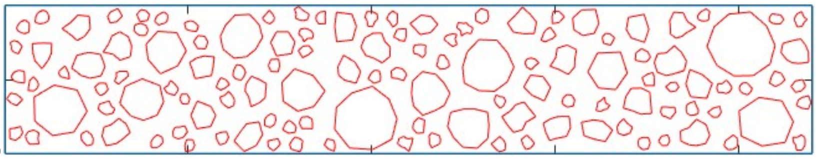

(c)

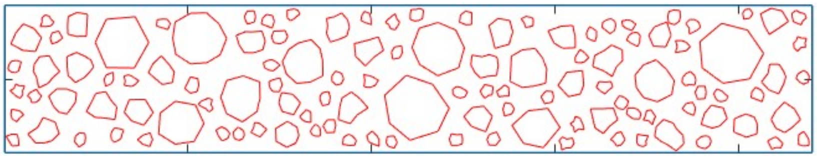

(d)

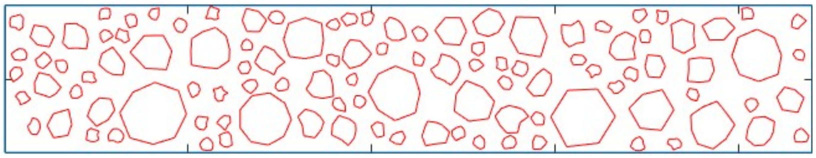

(e)

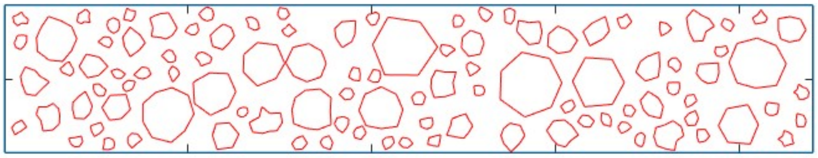

Fig.S2 Two-dimensional digital specimen: (a) No. 1 mix ratio two-dimensional digital specimen, (b) No. 2 mix ratio two-dimensional digital specimen, (c) No. 3 mix ratio two-dimensional digital specimen, (d) No. 4 mix ratio two-dimensional digital specimen, and (e) No. 5 mix ratio two-dimensional digital specimen.

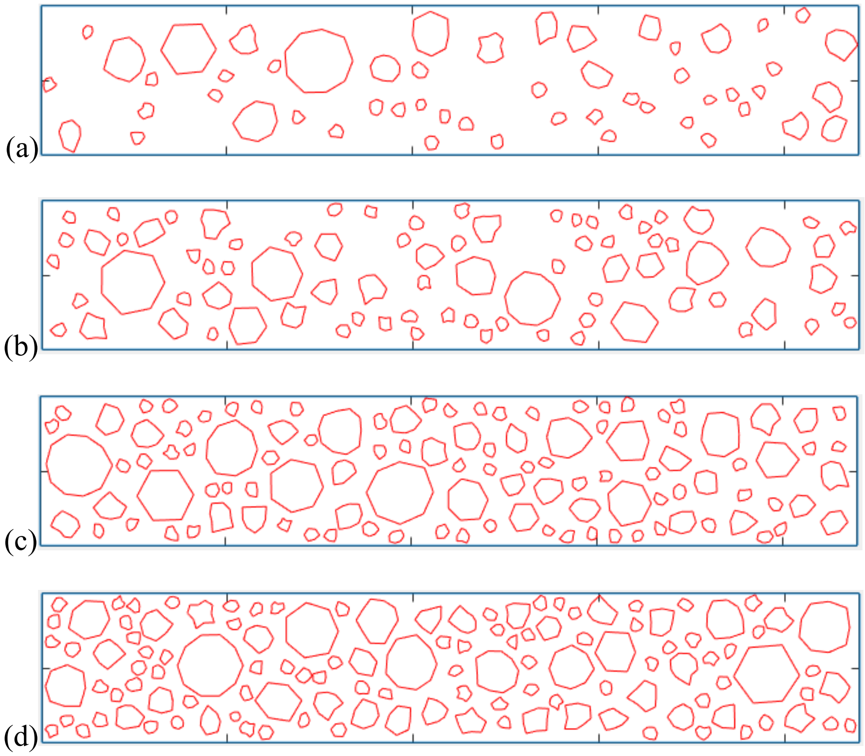

Fig.S3 (a) Two-dimensional digital specimen with 20% coarse aggregate content, (b) Two-dimensional digital specimen with 30% coarse aggregate content, (c) Two-dimensional digital specimen with 40% coarse aggregate content, and (d) Two-dimensional digital specimen with 50% coarse aggregate content.

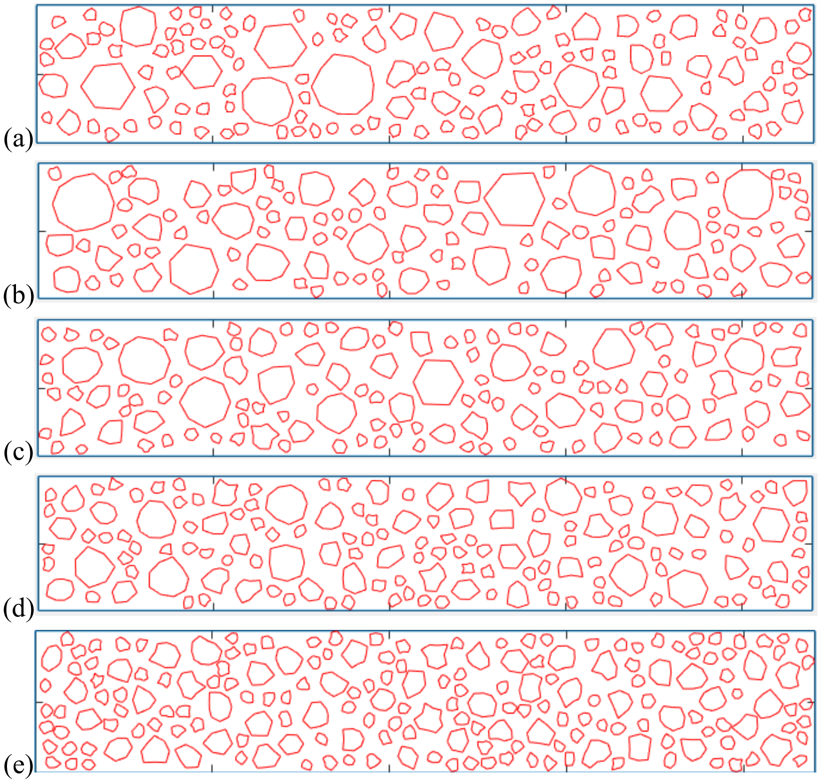

Fig.S4 (a) A two-dimensional digital specimen with a maximum particle size of 19mm, (b) A two-dimensional digital specimen with a maximum particle size of 16mm, (c) A two-dimensional digital specimen with a maximum particle size of 13.2mm, (d) A two-dimensional digital specimen with a maximum particle size of 9.5mm, and (e) A two-dimensional digital specimen with a maximum particle size of 4.75mm.

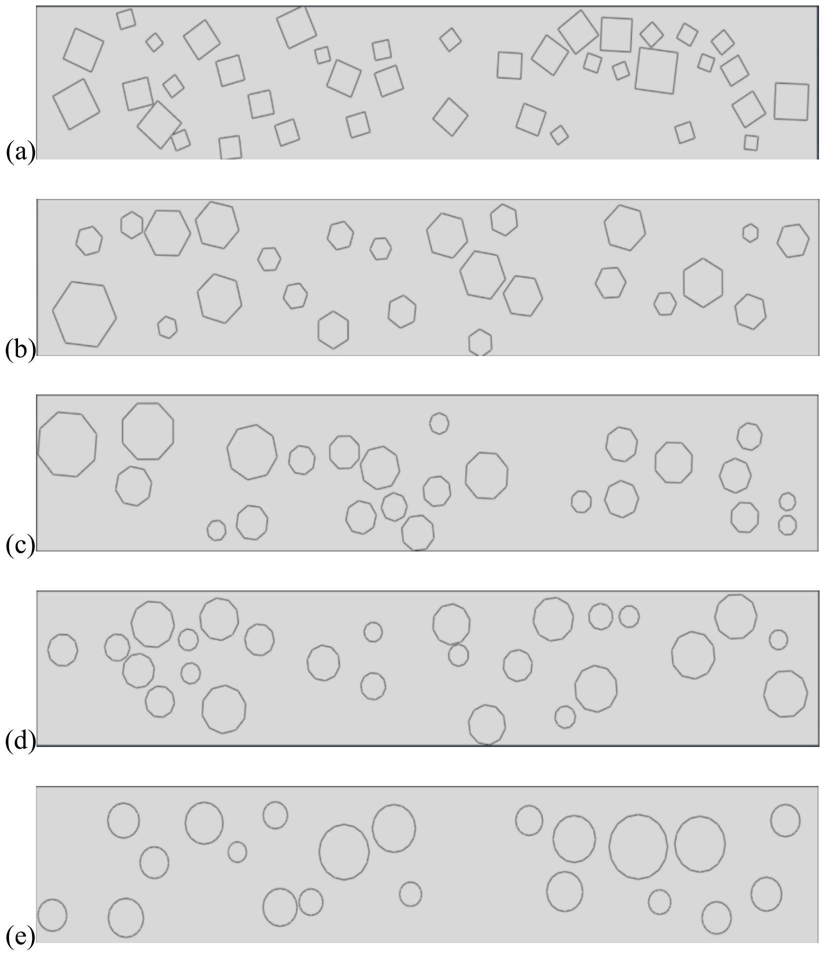

Fig.S5 (a) Two-dimensional digital specimen of regular quadrilateral coarse aggregate, (b) Two-dimensional digital specimen of regular hexagonal coarse aggregate, (c) Two-dimensional digital specimen of regular octagonal coarse aggregate, (d) Two-dimensional digital specimen of regular decagonal coarse aggregate, and (e) Two-dimensional digital specimen of round coarse aggregate.

Table S1 Preferred mix ratio parameter table

greatest particle size	gradation exponents	Filler dosage F	Percentage of mass of each mineral grade %					
mm	R	%	19-13.2 mm	13.2-9.5 mm	9.5-4.75 mm	4.75-2.36 mm	2.36-0.075 mm	<0.075 mm
19	0.5	9	16.17	12.27	20.11	14.33	28.12	9

Table S2 Three-dimensional asphalt concrete grading table

Gradation type	Maximum particle size	gradation index	Filler dosage F	Percentage of mass of each mineral grade %				
	mm	R	%	19-16 mm	16-13.2 mm	13.2-9.5 mm	9.5-4.75 mm	4.75-2.36 mm
1	19	0.3	9	5.65	5.98	9.46	17.13	14.03
2	19	0.4	9	6.79	7.06	10.89	18.75	14.32
3	19	0.5	9	7.99	8.17	12.27	20.11	14.33
4	19	0.6	9	9.25	9.28	13.59	21.19	14.09
5	19	0.7	9	10.53	10.38	14.81	21.99	13.63

Table S3 Two-dimensional asphalt concrete grading table

Gradation type	greatest particle size	gradation index	Filler dosage F	Percentage of mass of each mineral grade %				
	mm	R	%	19-16 mm	16-13.2 mm	13.2-9.5 mm	9.5-4.75 mm	4.75-2.36 mm
1	19	0.3	9	10.80	11.45	18.11	32.79	26.85
2	19	0.4	9	11.74	12.22	18.83	32.43	24.77
3	19	0.5	9	12.71	13.00	19.52	31.98	22.79
4	19	0.6	9	13.72	13.77	20.16	30.44	20.90
5	19	0.7	9	14.76	14.55	20.76	30.82	19.11

Table S4 Percentage of quantity passing through each sieve (mm)

Maximum particle size (mm)	Percentage of sieve residue (%) for each sieve hole (mm)					
	19	16	13.2	9.5	4.75	2.36
19	100	88.43	76.60	58.84	29.74	0
16	100	100	86.45	66.10	32.76	0
13.2	100	100	100	76.09	36.91	0
9.5	100	100	100	100	46.86	0
4.75	100	100	100	100	100	0

Table S5 Angularity index of different shapes of coarse aggregates

Geometry	regular quadrilateral	regular hexagon	regular octagon	regular decagon	orbicular
sharpness index	1.273	1.103	1.055	1.034	1.000
